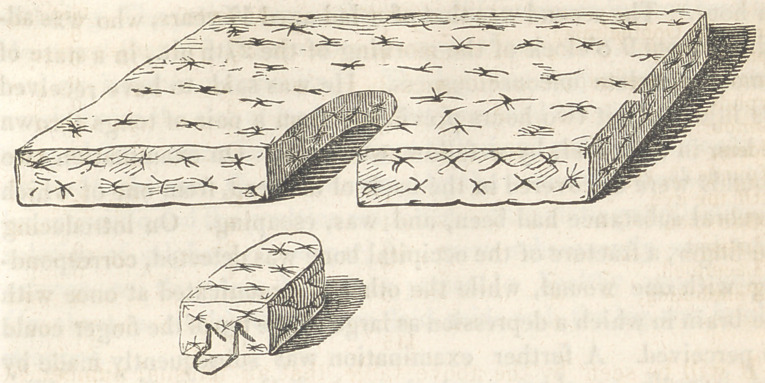# Pennsylvania Hospital

**Published:** 1849-10

**Authors:** James J. Levick

**Affiliations:** Resident Physician; Pennsylvania Hospital


### Pennsylvania Hospital.—*Surgical Wards.—Service of* Dr. Fox.

*Cases discharged from August 1st to September 15th, 1849.*

| | Cured. | By request. | Died. |
|---|---|---|---|
| Abscess, - - - - - | 1 | 1 | 0 |
| Bunyon, - - - - - | 1 | 0 | 0 |
| Burn, - - - - - | 3 | 0 | 0 |
| Cancer of lip, - - - - | 2 | 0 | 0 |
| Do. breast, - - - | 0 | 1 | 1 |
| Closure of pupil, - - - | 1* | 0 | 0 |
| Compression of brain, - - | 0 | 0 | 1 |
| Concussion of brain, - - | 1 | 0 | 0 |
| Contusions, - - - - | 5 | 0 | 0 |
| Coxalgia, - - - - - - | 0 | 1 | 0 |
| Disease of knee joint, - - | 3† | 0 | 0 |
| Do. skin, - - - - | 2 | 0 | 0 |
| Dislocation of ankle, - - - | 1 | 0 | 0 |
| Do. thigh, - - - | 1 | 0 | 0 |
| Compound dislocation of elbow, | 1‡ | 0 | 0 |
| Epulis, - - - - - - | 1 | 0 | 0 |
| Fistula in ano, - - - - | 1 | 0 | 1§ |
| Fractures 32, simple 26, viz.: | | | |
| Arm, - - - - - - | 8 | 0 | 0 |
| Clavicle, - - - - - | 3 | 0 | 0 |
| Fore arm, - - - - - | 5 | 1 | 0 |
| Leg, - - - - - | 2 | 0 | 1‖ |
| Metacarpus, - - - - - | 1 | 0 | 0 |
| Metatarsus, - - - - - | 1 | 0 | 0 |
| Rib, - - - - - | 1 | 0 | 0 |
| Scapula, - - - - - | 0 | 1 | 1 |
| Thigh, - - - - - - | 1 | 0 | 0 |
| Fractures, compound, 6 viz.: | | | |
| Leg, - - - - - - | 2 | 0 | 1‖ |
| Skull, - - - - - - | 0 | 0 | 2 |
| Upper jaw, - - - - - | 1 | 0 | 0 |
| Gonorrhœa, - - - - - | 8 | 0 | 0 |
| Hydrocele, - - - - - | 2 | 0 | 0 |
| Inflamed arm, - - - - - | 2 | 0 | 0 |
| Do. eye, - - - - - | 1 | 0 | 0 |
| Do. foot, - - - - | 1 | 1 | 0 |
| Do. hand, - - - - | 4 | 0 | 0 |
| Do. knee, - - - - - | 4 | 0 | 0 |
| Do. leg, - - - - - | 1 | 1 | 0 |
| Injury of spine, - - - | 1 | 1 | 1 |
| Iritis, - - - - - - | 1 | 0 | 0 |
| Necrosis, - - - - - | 1 | 0 | 0 |
| Orchitis, - - - - - | 1 | 0 | 0 |
| Phymosis (Conginital), - - | 1 | 0 | 0 |
| Polypus of ear, - - - - | 1 | 0 | 0 |
| Scrofula, - - - - - - | 1 | 0 | 0 |

* By operation for artificial pupil. † One by amputation.

‡ By amputation. § Of phthsis pulmonalis. ‖ Of mania a potu.

| | Cured. | By request. | Died. |
|---|---|---|---|
| Strumous Ophthalmia, - - | 1 | 0 | 0 |
| Sprain, - - - - - - | 1 | 0 | 0 |
| Stricture of urethra, - - - | 1 | 1 | 0 |
| Syphilis, - - - - - | 10 | 3 | 0 |
| Tumour, - - - - - - | 0 | 1 | 0 |
| Ulcers, - - - - - - | 7 | 0 | 0 |
| Wounds 22, viz.: | | | |
| Of the eye, - - - - | 1 | 0 | 0 |
| Gun shot, - - - - - | 1 | 0 | 1 |
| Incised, - - - - - | 7 | 2 | 0 |
| Lacerated, - - - - | 7 | 1 | 0 |
| Penetrating, - - - - | 0 | 1 | 1 |
| | 115 | 17 | 11 |

It will be seen by the above table, that an unusual number of fractures has been under treatment and discharged during the months of August and September. These accidents continue to increase, as I find by reference to the register that there have been received since the first of August up to the present time—some of which still remain in the house—forty-seven cases of broken bones.

The treatment of these has been that successfully pursued at the hospital for many years. Fractures of the clavicle, with the sling and pad of Dr. Fox; fractures of the condyles of the humerus, with the angular splint placed on the front of the arm, as described in July No. of the Examiner; fractures of the os femoris, with the apparatus of Dessault, as modified by Physick and Hutchinson.

Some inconvenience having resulted from the too yielding nature of the material of which the mattresses used in the wards are made, (ordinary curled hair,) Dr. Fox, while on duty, had one constructed which appears to possess many advantages over those heretofore used. The material employed is the husk or envelope of Indian corn, torn in shreds, and slightly twisted. The mattress is sewed closely, and affords a firm support to the body of the patient. In the centre of this, instead of the ordinary circular hole usually to be found over the bed pan, is an U shaped opening, extending to the outer side of the bed, to which is fitted a pad made of the same material as the mattress, and one inch less in thickness. On the under surface of this pad, a board an inch thick, and perfectly smooth, is bound, which slides readily over the corresponding part of the bedstead. A loop of binding is attached to the outside of the pad, to render its removal more easy. (*Vide cut.*)

The advantages possessed by this will be at once apparent to all who have been accustomed to the treatment of fractured thighs. In the ordinary mattress, a depression at the patient's buttocks raises the lower end of the upper fragment to such an extent as not unfrequently to render necessary the application of a paste-board splint on the front of the thigh; while the removal of the circular pad, even by the hands of careful nurses, is very liable to disturb the perfect apposition of the fragments. In this, when the pad is in its place, the patient lies on a perfectly horizontal plane; and if it be necessary to use the bed pan, the pad can be gently withdrawn by the outside loop without communicating the least disturbance to the fractured limb. To render it still firmer, it is proposed that the sides of the board should fit in a corresponding groove, either in the opening of the mattress, or one in the bedstead, as may be found most available. As the mattress is alike on both surfaces, it may of course be used for either thigh.

The two fatal instances reported, of fracture of the leg, were men of most intemperate habits, who received their injuries while intoxicated, and died of mania a potu a few days after their admission. The fracture of the scapula was in each instance the result of a fall from a great height. The first remained in the house but a few hours having been subsequently removed to his home. The second, a man who had fallen from the roof of a five story building, striking his head and shoulder in the fall, never recovered from the concussion. The compound fractures of the skull, both of which proved fatal, were first that of a colored man who had fallen from a height of three or four stories, and was brought to the hospital in a dying condition; he expired in less than an hour. The second was that of a lad aged 17 years, who was admitted about 9 o'clock of the morning of the 27th ult., in a state of almost complete unconsciousness. He was said to have received his injury about two hours previously from a pair of tongs thrown at him in a quarrel, by a fellow workman. On examination two wounds were discovered in the back of his head, from one of which cerebral substance had been, and was, escaping. On introducing the finger, a fracture of the occipital bone was detected, corresponding with one wound, while the other communicated at once with the brain in which a depression as large as the top of the finger could be perceived. A further examination was subsequently made by the attending surgeons, and the external wound having been enlarged, the injury to the brain was still more apparent; no loose bone could be found. As it was thought that the fragment might have been removed by the physician out of the house who had first seen him, and as no benefit could result from further exploration, the edges of the wound were brought together, and retained loosely by two stitches, and the cold water dressing applied. He continued in a state of partial stupor, rendering the exhibition of medicines impracticable; had several convulsions, and died on the 30th of the same month. A post mortem examination discovered the presence of pus on the dura mater, the brain highly inflamed, and in its substance a large excavation corresponding with the external wound, and extending to some distance in its vicinity. The brain having been cut open, a piece of bone fitting the orifice of the os occipitis was found deeply imbedded in its substance.

The dislocation of the femur was into the foramen ovale; it was reduced with the pullies after the manner recommended by Sir Astley Cooper; the man left the house, well, in sixteen days.

A more full history of this case, as well as that of a recovery from a bad compound fracture of the leg will probably be given in a future number.

A case in which the application of straps of adhesive plaster in the treatment of ulcers of the lower extremity,—Baynton's method as improved by Critchett,—was successfully used, will be briefly described. The patient, a farmer, aged 37 years, entered the hospital August 15, with an extremely indolent ulcer over the middle part of the shin bone, with uneven edges and much below the surface of the leg. The patient was at the time of his entrance in good health. He stated that it had been sore for more than a year, occasionally healing, but soon reappearing. The leg was very much swollen, nearly twice the size of its fellow, but tense and not pitting on pressure. After a warm bath he was put to bed and a flax-seed poultice applied to the part. On the following day the wound was touched with lunar caustic and the poultice reapplied. Stimulating applications, solution of sulphate of copper, &c., were subsequently used, and as soon as the ulcer began to present a healthy appearance, (in about a week) these were laid aside and the leg was firmly strapped.

The application of these straps was made as directed by Critchett in the Lancet of October, 1848, page 417. The first strap, about an inch wide, was applied by its centre low down on the back of the heel and then pressed by the palm of the hand along both sides of the foot. The second piece was applied in a similar manner under the heel, and carried up at right angles to the first, the third, which slightly overlapped it, was applied to the back of the heel above the first and brought forward as before mentioned; the fourth under the foot, &c. This was continued until the foot was covered; the straps were then carried up the leg as high as the knee. Over this an ordinary roller was firmly applied. The patient remained in bed for three days, by which time the size of the leg having diminished, rendering the straps somewhat loose, they were removed. The ulcer was found to be rapidly filling up. The same application was again made and the patient allowed to rise. Five days later it was again removed, and the ulcer was found to have cicatrized, the new skin being on a level with the old, or nearly so, and healthy in its appearance. The patient remained in the hospital a week longer, still wearing the straps and walking about as usual. No disposition to a return of the ulcer appeared, and the man left the house September 7th, perfectly well, having at his own request had the straps renewed immediately before going out, still wishing to wear that which, in his own words, was so pleasant, and gave so much strength to his leg.

The compound dislocation of the elbow, was the result of entanglement of the arm in machinery. The joint was opened and the parts shockingly lacerated. Amputation of the humerus was performed by Dr. Fox. The arm was found unusually vascular, and a very large number of ligatures was necessary. A piece of dry lint was interposed between the flaps to prevent too speedy union of the external parts, the cold water dressing applied at once, and continued during the treatment. The lint was removed four or five days subsequently, the ligatures came away safely, and the patient left the hospital in one month and two days after the operation.

JAMES J. LEVICK, Resident Physician.

*Pennsylvania Hospital, September* 15, 1849.

## Figures and Tables

**Figure f1:**